# The Adaptive Nature of the Bone-Periodontal Ligament-Cementum Complex in a Ligature-Induced Periodontitis Rat Model

**DOI:** 10.1155/2013/876316

**Published:** 2013-07-02

**Authors:** Ji-Hyun Lee, Jeremy D. Lin, Justine I. Fong, Mark I. Ryder, Sunita P. Ho

**Affiliations:** ^1^Division of Biomaterials and Bioengineering, Department of Preventive and Restorative Dental Sciences, University of California, San Francisco, CA 94143, USA; ^2^Division of Periodontology, Department of Orofacial Sciences, University of California, San Francisco, CA, USA

## Abstract

The novel aspect of this study involves illustrating significant adaptation of a functionally loaded bone-PDL-cementum complex in a ligature-induced periodontitis rat model. Following 4, 8, and 15 days of ligation, proinflammatory cytokines (TNF-**α** and RANKL), a mineral resorption indicator (TRAP), and a cell migration and adhesion molecule for tissue regeneration (fibronectin) within the complex were localized and correlated with changes in PDL-space (functional space). At 4 days of ligation, the functional space of the distal complex was widened compared to controls and was positively correlated with an increased expression of TNF-**α**. At 8 and 15 days, the number of RANKL(+) cells decreased near the mesial alveolar bone crest (ABC) but increased at the distal ABC. TRAP(+) cells on both sides of the complex significantly increased at 8 days. A gradual change in fibronectin expression from the distal PDL-secondary cementum interfaces through precementum layers was observed when compared to increased and abrupt changes at the mesial PDL-cementum and PDL-bone interfaces in ligated and control groups. Based on our results, we hypothesize that compromised strain fields can be created in a diseased periodontium, which in response to prolonged function can significantly alter the original bone and apical cementum formations.

## 1. Introduction

Mechanical loads as a result of chewing or biting promote homeostasis of the periodontal ligament (PDL) and that of the bone-PDL-cementum complex following development of the bone-PDL-tooth fibrous joint [1, 2]. Homeostasis of the joint is maintained by the PDL, which contains a heterogeneous population of cells, such as fibroblasts, osteoblasts, cementoblasts, and undifferentiated mesenchymal cells, and its entheses (attachment sites) with bone and cementum. These cells are sensitive to mechanical loads, which manifest into strains, and as a result can promote mineral formation or resorption. The cell-matrix and cell-cell interactions regulate continuous PDL turnover, modeling and remodeling of bone, and subsequent adaptation of primary and secondary cementum, including PDL-bone and PDL-cementum interfaces throughout their physiological function [2–5]. Accommodation of functional loads within the physiological range continues as long as the bone-PDL-cementum complex is protected from the oral environment by gingival epithelium and underlying connective tissues. However, the complex is a target for bacterial infection due to commensal microorganisms in the oral environment and the unique anatomical feature of this environment [6]. As a result, periodontal tissues are susceptible to bacterial invasion.

The relationship between host-microbial interactions and progression to periodontitis is thought to depend on a combination of the ecological shift in subgingival biofilm composition, genetic factors, and other extraneous influences [7]. Despite many extraneous factors, the “critical pathway” model for periodontal pathogenesis developed by Offenbacher [8] does not account for mechanical loads, that is, the effect of functional mechanics on disease progression in the load bearing joint of humans. Biomechanics and other functional perspectives on diseased joints are important areas of study since the altered homeostasis due to bacterial invasion can lead to pathological adaptation of unaffected tissues. Hence, the combinatorial effect of disease and functional (mechanical) loads on disease progression and joint adaptation is an important area of investigation. However, the challenge that lies in performing such studies is the limited access to specimens from humans. Hence, animal models in general and rat models in particular continue to be used extensively as an experimental system to elucidate the effect of diverse factors on periodontal pathogenesis and progression [9, 10]. Despite the extensive literature regarding the characteristics of periodontal progression in different animal models, few studies have described the effects of the disease from a biomechanical perspective.

Numerous human and animal studies have confirmed the infectious etiology of gingival inflammation [11–13], apical migration of the gingival epithelial attachment, and the eventual loss of underlying bone and connective tissue support [14, 15], that leads to clinical periodontitis. In this study, we employed the lipopolysaccharide-soaked rat ligature model to initiate the clinical features of periodontitis, namely inflammation and loss of PDL attachment and alveolar bone [16]. This model is an analog that stimulates the natural occurrence of periodontitis in humans—food impaction over time followed by acute and chronic inflammatory host responses [17–19]. Once disease is initiated, the complex is kept under continued function and the changes in clinical features can be correlated with the distribution of biochemical markers. In this study, the presence and distribution of the following biochemical markers in sections taken from hemimaxillae harvested at 4, 8, and 15 days of ligation and corresponding controls were assessed: receptor activator of nuclear factor *κ*B ligand (RANKL: a membrane-bound protein commonly found on PDL cells, osteoblasts, and T cells, that induce the expression of RANK on the surface of osteoclasts) [20, 21]; tartrate-resistant acid phosphatase (TRAP: an enzyme highly expressed by osteoclasts) [22]; tumor necrosis factor-alpha (TNF-*α*: a marker for systemic inflammation) [23], and fibronectin (FN: a glycoprotein adhesion molecule for fibroblasts that promotes extracellular matrix production) [24]. Moreover, changes in biochemical expressions and localization were correlated to morphological changes of the complex. Based on previous studies by others, morphological alterations of bone and cementum due to inflammation were associated with increased expressions of proinflammatory mediators [25–28], including a variety of cytokines, such as TNF-*α*, that are involved in inflammatory bone resorption [29]. RANKL, which is known to orchestrate osteoclastogenesis synergistically with TNF-*α* [30], was mapped throughout the bone-PDL-cementum complex and was expected to increase in inflamed periodontal tissue. In addition, active osteoclasts and correlative changes in biochemical distribution and alveolar bone resorption were identified. Finally, adaptive changes in secondary cementum marked by FN expression [31] were discussed within the context of joint function. Our overall hypothesis was that coronal degradation due to periodontitis can cause a significant change in the biomechanics of the complex and that the resulting adaptive effects may not be the same between bone and secondary cementum. The objective was to identify the adaptation of this load-bearing joint, that is, the bone-PDL-tooth complex, with the onset of periodontitis by mapping and correlating morphological changes of the tooth and the alveolar socket to key biomolecular expressions within the complex. 

## 2. Materials and Methods

To minimize the effects of environmentally induced periodontitis [32–34], 6-week-old male Sprague-Dawley rats were housed in a germ-free facility (Parnassus Services Building) and fed a hard pelleted diet for the duration of the study. All animals included in this study were housed in pathogen-free conditions in compliance with the guidelines of the Institutional Animal Care and Use Committee (IACUC) of UCSF and the National Institute of Health (NIH).

### 2.1. Induction of Periodontitis Using an *In Vivo* Rat Ligature Model

**Figure 1:**
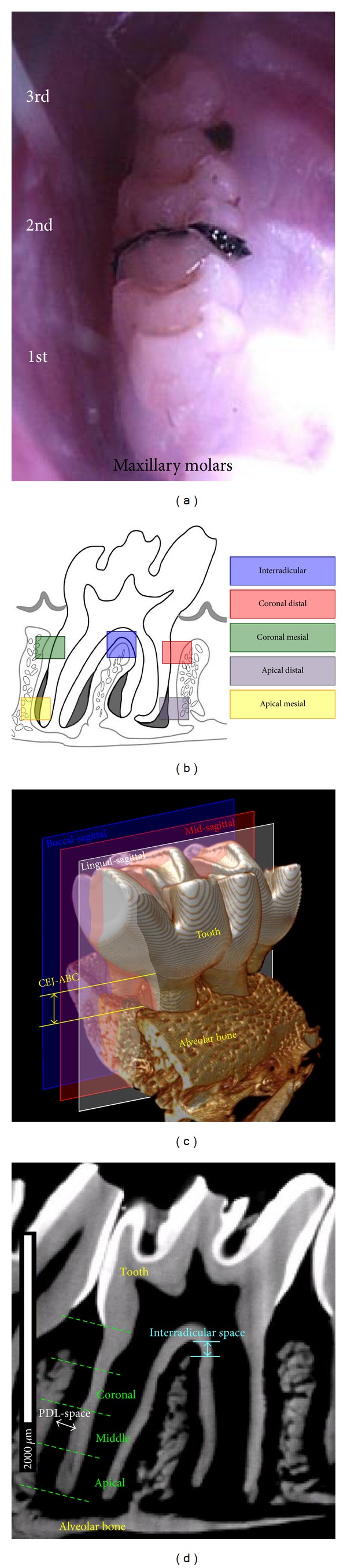
*In vivo* rat ligature model for the induction of acute periodontitis. (a) Photograph illustrates lipopolysaccharide (LPS) soaked 4–0 braided silk threads in the diastemata flanking left/right second maxillary molars. Controls were flossed in the same interproximal regions. (b) Schematic illustrates the targeted regions of the fibrous joint within the study. (c) 3D tomogram illustrates the lingual-sagittal, mid-sagittal, and buccal-sagittal 2D virtual sections through a second maxillary molar used for morphometrics. Anatomical landmarks used to measure alveolar bone crest recession (CEJ-ABC) are indicated. (d) 2D virtual section illustrates anatomical landmarks to measure interradicular distance and PDL width. Division of the bone-PDL-cementum complex into coronal, middle, and apical sections for PDL-space measurements is also illustrated.

4–0 silk suture threads soaked in 1 mg of lipopolysaccharide (LPS) from *Escherichia coli* serotype 055:B5 (Sigma-Aldrich, USA) per 1 mL of 1x Tris buffer were used to induce periodontitis (*N* = 5 per time point). Threads were placed between the first and second molars and the second and third molars of both maxillae (Figure 1(a)). Molars were religated every 2-3 days to ensure retention. Control rats (*N* = 5 per time point) were flossed every 2-3 days with 4–0 silk ligatures without LPS. Rats were euthanized after 4, 8, and 15 days of ligation. Maxillae were harvested and hemisected. Right hemimaxillae were stored in 70% ethanol for micro-XCT analysis. Left hemimaxillae were fixed in 4% paraformaldehyde (PFA) at room temperature overnight for histology.

### 2.2. Histological Analysis of Cytokine Expressions Using Immunohistology, Identification of Bone Resorption through TRAP(+) Osteoclasts, and Observation of Changes in Collagen Birefringence

Following fixation, intact hemimaxillae (*N* = 5) were decalcified in 0.5 M ethylenediaminetetraacetic acid (EDTA) solution for 3 weeks. The EDTA solution was changed every 3 days. Specimens were then dehydrated through 80%, 95%, and 100% Flex Alcohol (Richard-Allan Scientific, Kalamazoo, MI USA) before embedding in paraffin (Tissue Prep-II, Fisher Scientific, Fair Lawn, NJ USA). Embedded specimens were sagittally sectioned on a rotary microtome (Reichert-Jung Biocut, Vienna, Austria) using a disposable steel blade (TBF Inc., Shur/Sharp, Fisher Scientific, Fair Lawn, NJ USA). Paraffin serial sections were mounted on Superfrost Plus microscope slides (Fisher Scientific, Fair Lawn, NJ). Sections were deparaffinized with xylene and rehydrated through a descending ethanol series of 100%, 95%, and 80% ethanol before further use.

#### 2.2.1. Immunostaining for RANKL, FN, and TNF-alpha

The immunofluorescence staining protocol used for RANKL and FN was based on a previously described protocol [35]. In brief, deparaffinized sections were digested with trypsin (Sigma-Aldrich, St. Louis, MO, USA) at 37°C. Following washing, the specimens were incubated in blocking buffer (3% goat serum, 0.1% BSA in 1x PBS) and then in primary antibodies of polyclonal rabbit anti-RANKL (Santa Cruz Biotechnology Inc., sc-9073, Santa Cruz, CA, USA) and monoclonal mouse anti-FN (Santa Cruz Biotechnology Inc., sc-8422, Santa Cruz, CA, USA) diluted to 1 : 50 in blocking buffer. Slides were stored at 4°C followed by washing with 0.1% Tween-20 in PBS (PBST) and were incubated with secondary antibodies. AlexaFluor 594 goat anti-rabbit (Invitrogen, A-11012, Carlsbad, CA, USA) and AlexaFluor 488 goat anti-mouse (Invitrogen, A-11029, Carlsbad, CA, USA) were used to label polyclonal rabbit anti-RANKL and monoclonal mouse anti-FN at 1 : 300 (diluted in blocking buffer), respectively. Sections were washed with PBST and then stained with 1 : 10,000 trihydrochloride trihydrate (Invitrogen, Carlsbad, CA, USA) for ten minutes in the absence of light. Slides were rinsed twice with PBS and mounted using Fluoro-Gel (Electron Microscopy Sciences, Hatfield, PA, USA). Stained sections were visualized using Eclipse E800 fluorescent microscope (Nikon Inc., Melville, NY). TRITC filter (540–565 nm) was used to excite AlexaFluor 594 (abs. 590 nm, emit. 617 nm), FITC filter (465–495 nm) to excite AlexaFluor488 (abs. 495 nm, emit. 519 nm), and DAPI filter (340–380 nm) to excite trihydrochloride trihydrate (abs. 358 nm, emit. 461 nm). Images were stitched using Microsoft Research Image Composite Editor (Microsoft Corporation, Redmond, WA, USA). 

For quantitative analysis for RANKL, the number of RANKL(+) cells in two 125 *μ*m square regions around the surface of alveolar bone crest was counted using Image J (v1.44p, National Institute of Health, USA). Group means (±standard deviation) were calculated. A two-way analysis of variance (ANOVA) was used to analyze the effect of time and experimental conditions. A follow-up post-hoc test was used to analyze the differences between groups. *P* < 0.05 was taken to indicate significance. For comparative evaluation of FN, a 300 *μ*m line plot spanning dentin, secondary cementum, PDL, and alveolar bone, were generated for immunofluorescence micrographs of each group, and gradients of FN intensities were mapped using Image J.

3,3′-Diaminobenzidine (DAB) staining for detection of TNF-*α* was performed on serial sections. Endogenous peroxidases were deactivated with 80% methanol and 0.6% H_2_O_2_. Following antigen retrieval, sections were incubated with normal serum for 30 min to prevent nonspecific binding. For cytokine detection, the primary antibody (goat polyclonal anti-rat TNF-*α*, sc-1350 Santa Cruz Biotechnology, Inc., Santa Cruz, CA. USA) was applied on the sections at a dilution of 1 : 100 in PBS and incubated overnight at room temperature. The sections were incubated for 15 min at room temperature with the secondary antibody (biotinylated rabbit anti-goat IgG antibody, PK-6105, Vector Labs, Burlingame, CA. USA). Antigen-antibody complexes were visualized with DAB tetrachloride solution (Sigma, D3939, St. Louis, MO, USA), washed in distilled water, counterstained with hematoxylin Gill (3X) (Fisher Scientific, Kalamazoo, MI, USA), and rinsed in running water. Finally, the sections were dehydrated in ascending concentrations of alcohol, cleared with xylene, and mounted. Negative controls were obtained by substitution of the primary antibodies with normal goat serum. Lung tissues harvested from the same animals were used as positive controls. The sections were evaluated by a single examiner, who was blinded to the treatment assignment using a light microscope (BX 51, Olympus America Inc., San Diego, CA, USA).

#### 2.2.2. Resorption by Mapping TRAP(+) Osteoclastic Cells

 Tartrate-resistant acid phosphatase (TRAP) staining for osteoclasts was performed by treating rehydrated specimens with 0.2 M acetate buffer, a solution of 0.2 M sodium acetate, and 50 mM L-(+)-tartaric acid (Sigma-Aldrich, St. Louis, MO, USA). After 20 minutes of incubation at room temperature, naphthol AS-MX phosphate and fast red TR salt were added, followed by incubation at 37°C for 1 hour with close monitoring under the microscope after the first half hour to monitor the development of a bright red staining for osteoclastic activity. The stained sections were washed in deionized water, counterstained with hematoxylin, and mounted with Immu-Mount (ThermoScientific, Fremont, CA, USA) for subsequent examination under light microscopy. TRAP(+) stained regions were categorized based on location: the segment from alveolar crest (defined as the curved surface connecting the mesial and distal faces of the alveolar bone proper) to the starting point of secondary cementum as the coronal segment, and from secondary cementum to the apex as the apical segment. Criteria for identification of osteoclasts were TRAP(+) staining with greater than three nuclei [36]. The number of osteoclasts within each region was manually counted along the PDL-bone perimeter using Image-Pro Plus v6.0 data acquisition software (Media Cybernetics, Inc., Bethesda, MD, USA) and ratios of osteoclast count to perimeter per mesial and distal location were calculated [37]. These ratios were tested for statistical differences between control and ligature groups and across time points using two-way ANOVA followed by post-hoc tests to analyze the difference between the groups. Differences with *P* < 0.05 were considered significant.

#### 2.2.3. Collagen Birefringence Using Picrosirius Red (PSR) Stain

 Deparaffinized sections were stained with Sirius red F3B (C.I. 35782) and picric acid (American MasterTech Scientific Co., Lodi, CA, USA). Stained sections were analyzed with a light microscope and Image-Pro Plus. Polarized light was used to enhance the birefringence of collagen to illustrate changes in collagen fiber orientation and birefringence intensity throughout the complex [38, 39].

### 2.3. Changes in Morphometrics of the Bone-PDL-Cementum Complex Using Micro-X-Ray Computed Tomography (*μ*-XCT)

Macroscale structural analysis of intact right hemimaxillae (*N* = 5 each group) was performed using micro- X-ray tomography (*μ*-XCT, Micro XCT-200, Xradia, Inc., Pleasanton, CA, USA) at 2x magnification. X-ray imaging was performed on specimens using a tungsten anode with a setting of 75 KVp at 6 W at binning 2 and quartz silica (SiO_2_) filter designed specifically for biological specimens. Specimens were scanned while immersed in 70% ethanol with the second molar centered in the field of view. 2000 projections were collected at an exposure time of 9–14 s for each projection.

Tomograms were reconstructed (XMReconstructor v8.1.6599, Xradia Inc., Pleasanton, CA, USA) and 2D virtual sections were generated (Xradia 3D viewer v1.1.6, Xradia Inc., Pleasanton, USA) to complete the following measurements using Image J. To analyze the progression of periodontitis through the time points, three sagittal sections containing apical foramen were made through each specimen: in the mesiodistal direction through (1) both buccal roots, (2) both lingual roots, and (3) the interradicular region (Figure 1(c)). Alveolar bone crest (ABC) resorption was determined by measuring the distance from the cementoenamel junction (CEJ) to the adjacent ABC of the second maxillary molar along mesial and distal roots (Figure 1(c)). The width of the PDL-space surrounding the second maxillary molar was determined using the aforementioned buccal and lingual sagittal sections (*N* = 3 each group). Mesial and distal roots were divided into fourths from the CEJ to the root apex (Figure 1(d)). From the three apical fourths, five PDL-space measurements from the alveolar bone to the root cementum per quarter section were measured using Image J. Interradicular PDL spaces (or interradicular distances) measured using the midsagittal section as the distance from the crest of the interradicular bone to the molar root furcation (Figure 1(d)). All statistical analyses for significant differences in morphometrics across and within time points were performed using two-way ANOVA combined with post-hoc tests. Differences with *P* < 0.05 were considered significant.

## 3. Results

*The Naturally Occurring Tension and Compression Fields in a Rat Bone-PDL-Tooth Complex *[35]. It should be noted that in a rat the distal side is more prone to mineral resorption while the mesial side is prone to mineral formation. From a biomechanical perspective, this is due to the tensile strains in the mesial side compared to reactionary compressive strains on the distal side. As a result, mineral is formed on the mesial side of the alveolar socket, while mineral is resorbed on the distal side of the same alveolar socket, thus maintaining a uniform functional space within the bone-PDL-tooth fibrous joint. 

### 3.1. Changes in the Expressions of Biochemical Markers within the LPS Soaked Ligature-Induced Periodontitis Model

#### 3.1.1. RANKL Expression in the Complex

**Figure 2:**
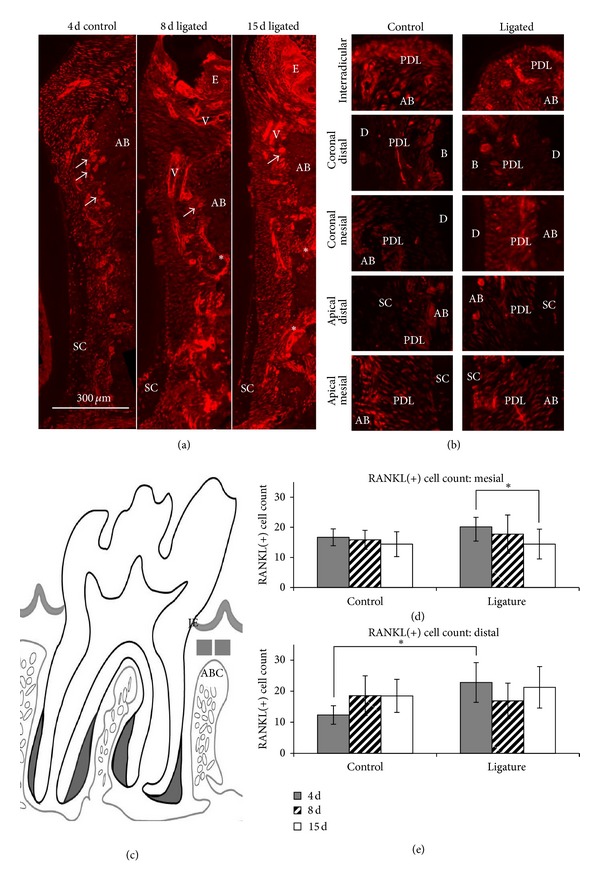
Identification of RANKL using immunofluorescence. (a) Representative micrographs illustrate immunofluorescence of antibodies against RANKL at 4-day control and 8- and 15-day ligated groups. Note RANKL expression around the vasculature (V) and endosteal spaces (asterisks). Multinucleated osteoclast-like cells were also observed at the PDL-bone interface (white arrows). (b) Higher magnification micrographs show RANKL immunofluorescence in local regions of the complex at 15 days of ligation. (c) Schematic of rat periodontal tissue (mesiodistal section) with gray boxes (125 *μ*m × 125 *μ*m) that indicate target areas used to count RANKL(+) cells. (d, e) Bar graphs illustrate RANKL(+) cell count within specified target areas between control and ligated groups on mesial (d) and distal (e) sides. *Statistically significant difference at 95% confidence interval was observed. Junctional epithelium (JE), epithelium (E), periodontal ligament (PDL), alveolar bone (AB), alveolar bone crest (ABC), dentin (D), and secondary cementum (SC).

Intense expression of RANKL in endosteal spaces of alveolar bone and vasculature in PDL was detected (Figure 2(a)) in the ligated group. In addition, osteoclasts in resorption pits at both PDL-bone and PDL-cementum attachment sites strongly expressed RANKL (Figure 2(b)). 

To correlate RANKL expression with osteoclastic activity, RANKL(+) cell number was counted in subepithelial connective tissue (CT) near the alveolar crest (Figures 2(c)–2(e)). RANKL(+) cell count interestingly exhibited advancing trends to TRAP(+) staining. On the mesial side (Figure 2(d)), the number of RANKL(+) cells in the ligated group was greater than that in the control group. However, the number of RANKL(+) cells was reduced with time in both groups, showing a significant decrease in ligated group from 4 days to 15 days (*P* < 0.05). On the distal side (Figure 2(e)), there was a significant elevation of RANKL(+) cells in the ligated group compared to the control group (*P* < 0.05) at 4 days, but a decrease was observed at 8 days. A higher number of RANKL(+) cell count was identified on the distal side compared to mesial side of the ligated group and distal and mesial sides of the control group (Figures 2(d) and 2(e)). 

#### 3.1.2. Osteoclastic Activity in the Complex

**Figure 3:**
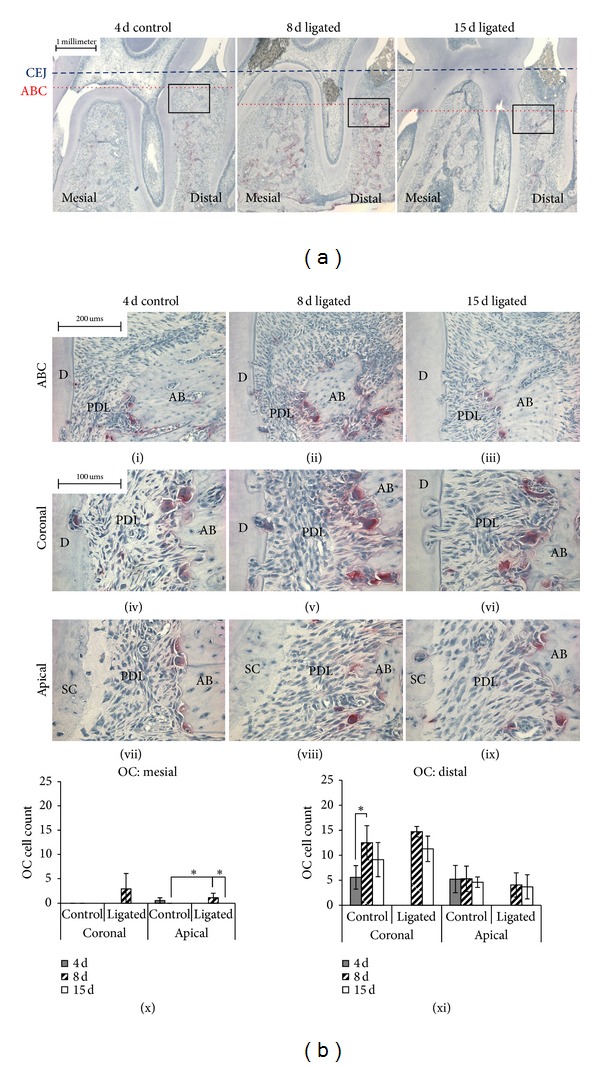
Alveolar bone resorption through TRAP(+) osteoclast identification. (a) Mesiodistal histological sections illustrate TRAP(+) cells on distal surfaces. The relative height of the alveolar bone crest (ABC) in relation to the cementoenamel junction (CEJ) is shown to decrease with duration of ligation. (b) Magnified images of 3A show alveolar bone crest (ABC), coronal, and apical regions of distal surfaces across time points and between control and ligated complexes (i–ix). The number of multinucleated osteoclasts (OC) located along the bone perimeter was counted in coronal and apical root segments on mesial (x) and distal (xi) sides. *Statistically significant difference at 95% confidence interval was observed. Alveolar bone (AB), periodontal ligament (PDL), dentin (D), and secondary cementum (SC).

Morphological changes were also evaluated in mesiodistal stained sections, and increased alveolar bone resorption with time was observed (Figure 3(a)). Consistent with *μ*-XCT morphometric analysis, the CEJ-ABC distance was higher in ligated groups (Figures 3(a) and 5(a)(i)). Multinucleated TRAP(+) cells were observed in endosteal and bone marrow spaces, irrespective of experimental conditions or time points (Figure 3(b)(i–ix)). Active osteoclasts at the PDL-bone interface were also found on the alveolar crest in the 8-day ligated group (Figure 3(b)(ii)). Quantification of TRAP(+) cells showed preferential distal localization regardless of experimental condition or time point (Figures 3(b)(x) and 3(b)(xi)).

Data for the 4-day ligated group was discarded due to inadequate samples. TRAP(+) cells showed preferential distal localization regardless of experimental condition or time point (Figures 3(b)(x) and 3(b)(xi)) and peaked at 8 days except in apical segments on the distal side. On the mesial side, osteoclastic activity at 8 days was significantly greater than that observed at 4 days in coronal segments and that of the 15-day ligated group in apical segments (Figure 3(b)(x)). Within control groups, no significant increase or decrease was seen in both coronal and apical segments. However, the mesial osteoclastic activity of ligated groups significantly increased at 8 days in apical segments compared to respective control groups before decreasing at 15 days (Figure 3(b)(x)). On the distal side, osteoclastic activity both at 8 days and 15 days was significantly greater than that of the 4-day control group in coronal segments when the 4-day control group was used as the reference (data not shown). At both 8 and 15 days, ligated groups showed an increase in osteoclastic activity in coronal segments compared to corresponding control groups, while osteoclastic activity in ligated groups decreased in apical segments (Figure 3(b)(xi)). Between 8 and 15 days, the distal osteoclastic activity of ligated groups decreased in both coronal and apical segments (Figure 3(b)(xi)).

#### 3.1.3. Immunohistochemical Localization of TNF-*α*

**Figure 4:**
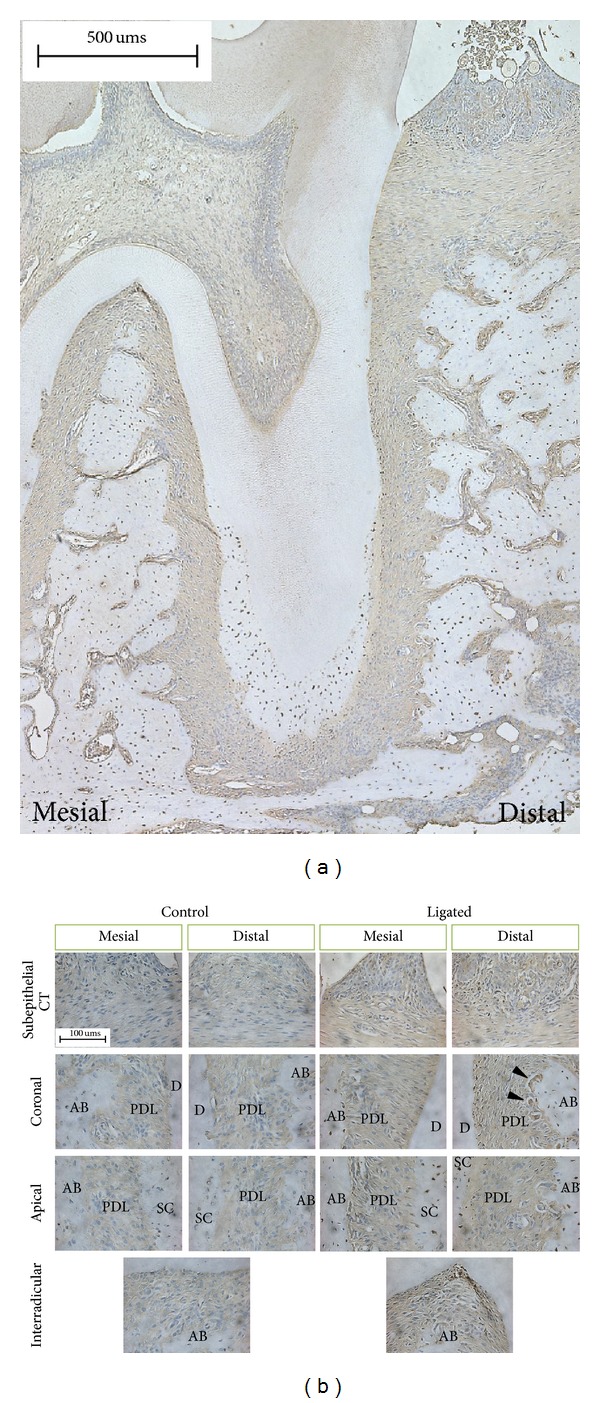
Immunohistochemical staining for identification of TNF-*α*. (a) Representative light micrograph of the complex at 15 days of ligation illustrates localization of TNF-*α*. (b) Representative images illustrate immunohistochemical localization of TNF-*α* at subepithelial connective tissue, coronal and apical PDL spaces, and interradicular PDL regions, according to experimental conditions. For example, note TNF-*α* expression at the distal coronal PDL-bone interface (black arrow heads). Connective tissue (CT), alveolar bone (AB), periodontal ligament (PDL), dentin (D), and secondary cementum (SC).

TNF-*α* was identified in the gingival epithelium, subepithelial CT, endosteal spaces, predentin, and secondary cementum (Figure 4(a)). Particularly, TNF-*α* expression in ligated groups was more intense than that of control groups at both mesial and distal PDL-cementum interfaces, the interradicular complex, and in endosteal spaces (Figure 4(b)). At a higher magnification, osteoclast-like cells were detected at PDL-bone interfaces in ligated groups (Figure 4(b), arrowheads).

### 3.2. LPS Soaked Ligature-Induced Periodontitis Rat Model Stimulated Significant Changes in Alveolar Bone Resorption, PDL-Space Width, Collagen Birefringence, and Fibronectin Expression 

#### 3.2.1. Alveolar Bone Resorption

**Figure 5:**
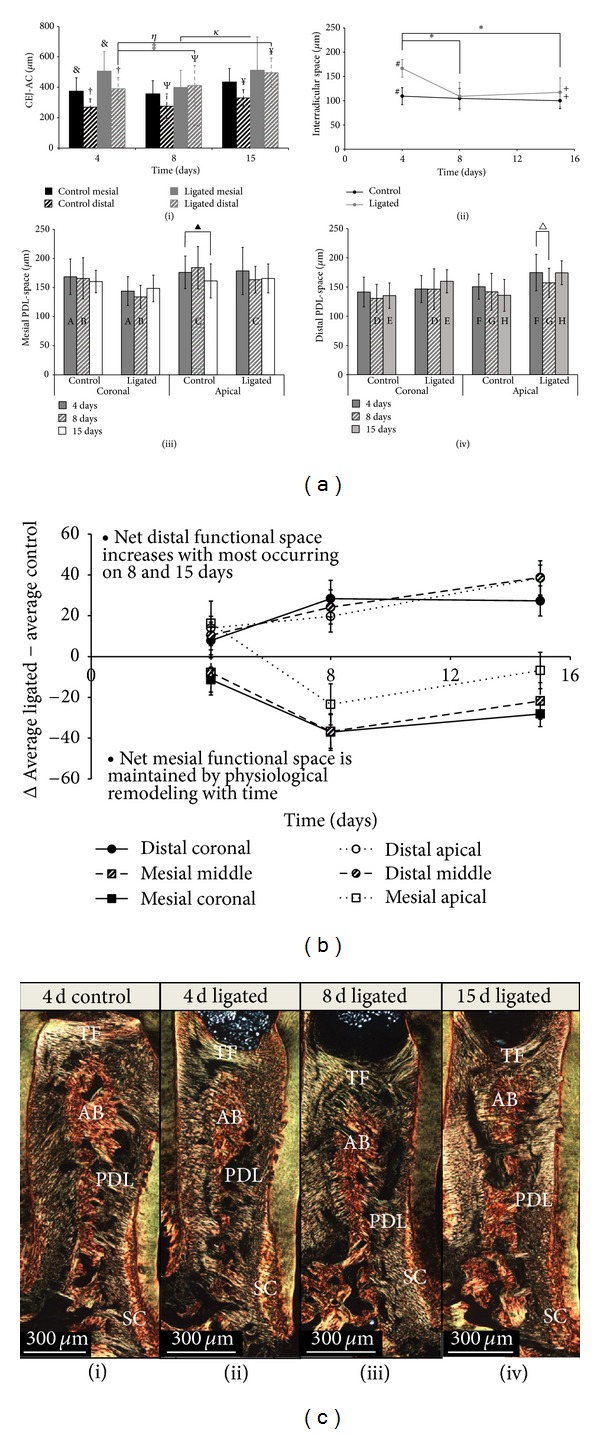
Morphometric comparisons of control and ligated bone-PDL-cementum complexes, including changes in collagen birefringence. (a) Comparisons of measurements in mesial and distal regions of the CEJ-ABC (i), the interradicular region (ii), and the PDL-space (iii, iv) between control and ligated complexes at 4, 8, and 15 days. Individual graphs were used to compare PDL-space measurements between coronal and apical anatomical locations within mesial (iii) and distal (iv) complexes. (b) The differences between average ligated and average control PDL-space measurements are plotted for each aforementioned anatomical location across time. (c) Histological sections show the distal complex stained with PSR and visualized under polarized light microscopy. Alveolar bone (AB), periodontal ligament (PDL), secondary cementum (SC), and transseptal fibers (TF). Symbols within plots indicate statistically significant differences at 95% confidence interval. ^&,†,Ψ,*¥*^Significant difference between control and ligated groups. ^*η*,‡,*κ*^Significant difference over time. ^#,+^Significant difference between control and ligated groups. *Significant difference over time. ^A,*Β*,C,D,E,F,G,H^Significant difference between control and ligated groups. ^▲,∆^Significant difference over time.

 Morphometric analysis using *μ*-XCT revealed that at all time points, the distance from CEJ to ABC (CEJ-ABC) was greater in ligated groups when compared to control groups in both mesial and distal regions (Figure 5(a)(i)). While at 4 days both mesial and distal regions of ligated groups exhibited significant increases in CEJ-ABC compared to controls, only the distal regions exhibited significant increases in corresponding comparisons. In mesial regions of both control and ligated complexes, CEJ-ABC exhibited a decreasing trend between 4 days to 8 days and an increase from 8 to 15 days; however, only the ligated mesial region exhibited a significant increase between 8 and 15 days (*P* < 0.05). In distal regions, the CEJ-ABC exhibited increasing trends in both control and ligated complexes with time, increasing significantly at 8 and 15 days when compared to 4 days (*P* < 0.05). The interradicular distance within the control complex decreased with time (Figure 5(a)(ii)). In contrast, the interradicular distance within the ligated complex decreased significantly between 4 and 8 days of ligation (*P* < 0.05) and then increased slightly after 15 days of ligation. Comparisons of the averages in ligated and control interradicular PDL-spaces at each time point showed that the ligated complex had a significantly greater interradicular PDL-space at 4 days (*P* < 0.05). This significant increase was not maintained at 8 days but was reestablished at 15 days. 

#### 3.2.2. PDL-Space

 PDL-space measurements showed differences in trends across time between control (Figure 5(a)(iii)) and ligated complexes (Figure 5(a)(iv)) in both coronal and apical regions. In all ligated regions, a decreasing followed by an increasing trend in PDL-space was found over time with a significant decrease in the distal apical region (*P* < 0.05). In contrast, control complexes did not exhibit this trend with the exception of the distal coronal region. Instead, between 8 and 15 days the PDL-space of both mesial and distal sides showed a decreasing trend with time in the control complex, with a significant decrease between 4 and 15 days at the mesial apical region (*P* < 0.05). When comparing the PDL-space between control and ligated complexes, it was interesting to note that all observed significant differences in the mesial regions occurred at the earlier time points of 4 and 8 days (*P* < 0.05). The significant differences in distal regions occurred mostly at later time points of 8 and 15 days (*P* < 0.05).

To separate the effect of treatment from that of development, the average control PDL-space was subtracted from the average treated PDL-space for each region (Figure 5(b)). The mesial apical and all distal PDL-spaces of the ligated complex increased at 4 days, while mesial coronal and mesial middle PDL-spaces decreased compared to controls. The mesial coronal, mesial middle, and distal apical regions, exhibited significant differences in PDL-space between ligated and control groups. Regardless of anatomical location at 8 days, the difference between ligated and control mesial PDL-spaces increased negatively and the difference between ligated and control of distal PDL-spaces increased positively with time relative to corresponding control regions (*P* < 0.05). As such, there was a significant difference in PDL-space between ligated and control groups in all regions. At 15 days, the distal PDL-spaces of ligated coronal regions remained greater than the corresponding control region but decreased in difference, while the distal PDL-spaces of ligated middle and apical regions increased in difference with corresponding control regions. Similarly, the mesial ligated regions still remained smaller than the PDL-spaces of controls; however, they decreased in difference with the corresponding control regions. Interestingly, only the distal regions exhibited significant differences between ligated and control PDL-space measurements at 15 days.

#### 3.2.3. Collagen Birefringence

 PSR birefringence changes were observed on the distal side depending on the duration of the insult (Figure 5(c), see Figure S1 and supplemental movies in Supplementary Material available online at http://dx.doi.org/10.1155/2013/876316). In coronal regions, the ligated complex showed compromised transseptal fiber integrity with decreased birefringence, unlike the observed high birefringence with straight collagen fibers in the control complex. In apical regions, the width of increased birefringence in the distal PDL-secondary cementum of the ligated complex widened across 4, 8, and 15 days (Figure 5(c)). It should be noted that birefringence is dependent on the angle between the PSR-stained specimen and the polarized light. For a complete representation of the above observation, supplemental movies are included.

#### 3.2.4. FN Expression at the PDL-Bone and PDL-Cementum Interfaces

**Figure 6:**
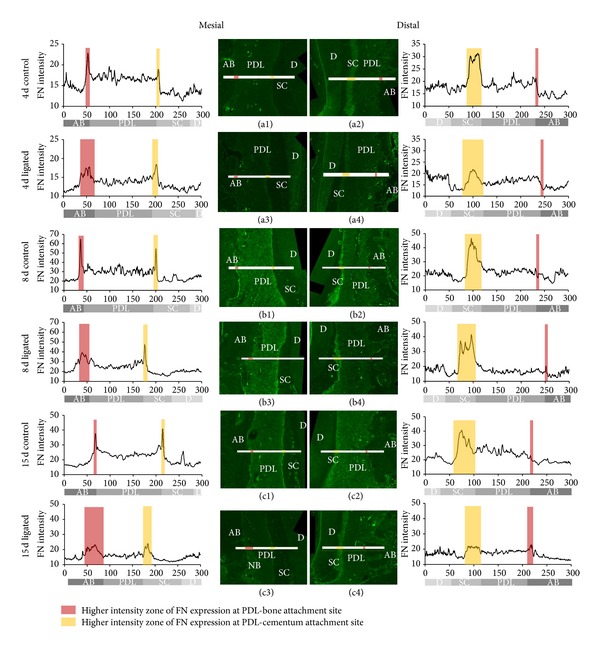
Line profiles and micrographs of immunolabeled fibronectin (FN). Representative micrographs illustrate FN immunofluorescence in apical regions of control and ligated complexes at 4, 8, and 15 days. The intensity of FN expression was measured along the anatomical locations indicated by the 300 *μ*m long bar. For the mesial complex, the *x*-axis of the 300 *μ*m long profile corresponds to alveolar bone (AB), periodontal ligament (PDL), secondary cementum (SC), and dentin (D). Note that the direction for the distal complex is reversed. (a1) 4-day control mesial complex, (a2) 4-day control distal complex, (a3) 4-day ligated mesial complex, (a4) 4-day ligated distal complex, (b1) 8-day control mesial complex, (b2) 8-day control distal complex, (b3) 8-day ligated mesial complex, (b4) 8-day ligated distal complex, (c1) 15-day control mesial complex, (c2) 15-day control distal complex, (c3) 15-day ligated mesial complex, and (c4) 15-day ligated distal complex. Dentin (D), secondary cementum (SC), periodontal ligament (PDL), alveolar bone (AB), new bone (NB).

 On the mesial side, FN expressions were illustrated as sharp peaks at both PDL-bone and PDL-cementum interfaces, regardless of the experimental condition and time point. However, the width of high FN intensity was wider in the ligated group compared to controls (Figures 6(a1), 6(a3), 6(b1), 6(b3), 6(c1), and 6(c3)). On the distal side, the display of FN over a larger width of cementum from the PDL-cementum attachment site was observed. On the other hand, only a sudden drop of intensity with no peak at the PDL-bone attachment site was shown (Figures 6(a2), 6(a4), 6(b2), 6(b4), 6(c2), 6(c4), and Figure S2).

## 4. Discussion

The results of this study are from a commonly used experimental rat model for periodontitis [40–44]. The impetus for choosing the ligature model is that it promotes scenarios similar to food impaction between teeth in humans. The ligatures between the 1st and 2nd molars and the 2nd and 3rd molars (Figure 1(a)) are speculated to promote equal and opposite forces, negating or minimizing effects due to the ligature itself. Additionally, LPS was used as a catalyst to accelerate coronal tissue degeneration by inducing hallmarks of periodontitis, such as alveolar crest resorption and PDL degeneration, thus promoting the loss of coronal tooth attachment. It should be noted that morphometrics, that is, PDL-space can increase or decrease. As a result, the functional space is either widened or narrowed, which in turn can alter overall biomechanics of the organ.

Based on observations from this study, it can be inferred that changes in biochemical expression and/or coronal-apical gradients resulting from inflammation could explain tissue degradation and a subsequent adaptive response driven by mechanobiology. However, future studies are needed to investigate potential bone formation and resorption effects during disease. This can be done by temporal mapping of mineral formation- and resorption-related events to elucidate PDL-cementum and PDL-bone interfaces under tension and/or compression using dynamic histomorphometry through a fluorochrome technique. Furthermore, our investigation is limited to the early stage of periodontitis, so a model for chronic periodontitis should be developed through observations made at longer time points by using the proposed endotoxin-ligature model. The longer ligation times would elucidate time-related mechanobiological effects on overall joint morphology and soft-hard tissue microstructure, including impairment of joint function—a topic currently under investigation.

The results of this study are discussed on the basis that both disease and function (continued mastication of hard pellets by the rats) have a concomitant effect on biochemical and subsequent morphological changes in the organ. The combined effect can result in positive or negative feedback, thus shifting organ function to impairment. Even though comprehensive studies have postulated a cause-and-effect relationship between bacteria and/or inflammatory cytokines and tissue destruction [44–51], they are spatially limited and describe only alveolar bone destruction. As a result, studies are needed to illustrate effects on the entire bone-PDL-cementum complex, in which the role of no one tissue dominates specifically when in function. 

Hence, the two concomitant effects that will be discussed will include: (1) inflammation-induced coronal degeneration as a result of LPS soaked threads, and (2) potential mechanobiological effects specifically in the apical regions of the tooth attachment apparatus due to coronal degeneration over time. To identify a host inflammatory response, we mapped expressions of RANKL, osteoclastic resorption, and TNF-*α*. Resulting adaptation was detailed through a change in morphology due to increased alveolar bone resorption and net changes in the PDL-space, coronal losses, apical increases in collagen birefringence within the complex, and a gain in FN expression specifically at the apical regions, that is, within secondary cementum and bone. These observed patterns are summarized in Figures S2 and S3. 

### 4.1. Inflammation-Related Biomolecular Expressions within the Complex

Ligature-induced inflammation is a host response to eliminate the harmful stimuli due to foreign body and bacterial colonization. The response triggers recruitment of immune cells from the blood into various vascularized connective tissues predominantly exposed to LPS and ligature impaction [27]. The recruited inflammatory cells in turn promote a biochemical cascade, causing proteinase-induced fibrinolysis, osteoclastogenesis, and activation of latent osteoclasts [16]. Formation and activity of osteoclasts *in vivo* are dependent on the expression of RANKL by osteoblastic or bone marrow stromal cells [52, 53]. As a result, we observed RANKL expression in endosteal spaces and vasculature of PDL. In this study, the coronal part of alveolar bone is most affected by LPS due to its proximity to the ligature, and as such the expression of RANKL(+) cells near the alveolar crest was quantified (Figures 2(d) and 2(e)). Interestingly, the observed RANKL(+) trend opposed the trend of TRAP(+) osteoclastic activity (Figures 3(b)(x) and 3(b)(xi)). In other words, the number of RANKL(+) cells increased at 4 days before decreasing and then rebounding at 15 days in the distal sides of ligated groups. It is well known that osteoclastogenesis involves a complex series of sequential steps, including RANKL-RANK signaling [54]. This could explain the observed trend of delayed osteoclastic activity, that is, TRAP(+) cells.

 Along with RANKL, TNF-*α* also plays a role in inflammatory bone resorption since TNF-*α* synergizes with RANKL to potentiate osteoclastogenesis [52, 55, 56]. The known mechanism by which TNF-*α* modulates bone destruction is related to RANKL stimulation of osteoclast differentiation through autocrine signaling [57]. The majority of *in vivo* studies show that TNF-*α* is mainly produced by activated macrophages and acts as an agonist or antagonist to advance or restrain alveolar bone resorption, respectively [47, 58–60]. From our observation, TNF-*α* was distributed throughout the periodontium (Figure 4(a)). This can be attributed to immune cells, the source of TNF-*α* that is recruited along the blood stream in highly vascularized PDL. The overall TNF-*α* expression was elevated in ligated groups, especially at PDL-primary cementum interfaces, the interradicular complex, and endosteal spaces. This implies that coronally placed ligatures introduced inflammation to the entire bone-PDL-cementum complex and was not just limited to the coronal area. Anatomically, TNF-*α* positive osteoclasts were predominant at the PDL-bone interface on the distal side in ligated groups. This observation was positively correlated with increased immunofluorescence of RANKL in conjunction with previous studies that have shown that TNF-*α* shares the mechanism by which RANKL exerts its osteoclastogenic effect [26, 57]. Additionally, TNF-*α* was localized to the junctional epithelium. It is plausible that TNF-*α* released extracellularly drifted into the epithelial layer, since the intercellular space in the junctional epithelium is comparatively wider and contains proportionately fewer desmosomes than in the oral epithelium [61], allowing cytokines to permeate epithelium. 

### 4.2. Changes in Morphometrics of the Complex, Common Hallmarks of Periodontitis

Ligated groups showed increased ABC and interradicular resorption compared to controls at 4 days. This observation is most likely caused by the early-stage host inflammatory events due to LPS as the primary stimulus. The argument can be further corroborated by the observation of a decrease in CEJ-ABC distance in mesial regions and a decrease in interradicular PDL-space at 8 days. However, the decrease in bone resorption despite heightened inflammation could have been caused by age-dependent bone modeling processes [42, 62] between 4 and 8 days as bone has a rapid rate of turnover. By 15 days, the resorptive effects of inflammation are speculated to overcome growth-related processes in ligated complexes, resulting in resumed increases of both mesial and distal CEJ-ABC, as well as interradicular PDL-space. It should be noted that within this observed effect is the effect of osteoclasts in controls due to natural distal resorption in rats [31, 62, 63]. However, the ligated complex included active osteoclasts on the alveolar crest in addition to the distal PDL-bone interface. Furthermore, the osteoclastic activity peaked at 8 days of ligation. This phenomenon can be explained in terms of the characteristics of inflammation induced with ligature placement, an acute and short-termed inflammation that resulted in the initial shift of equilibrium from bacterial endotoxin, that is, LPS, to host response. However, the osteoclastic activity was not maintained after 8 days, suggesting that the effect of inflammation induced by ligation was not sustained because (1) the location of ligature was relatively coronal, and (2) the host response that could have subsided as the local concentration of LPS was diffused [64]. This result is consistent with previous studies [65–69] and can be identified as remission of bone modeling following acute phase of inflammation. 

### 4.3. Resulting Early Mechanobiological Effects of a Diseased Fibrous Joint

Based on observed morphological differences (Figures 5(a) and 5(b)), we predict that the coronal degeneration could have shifted from physiological to nonphysiological, that is, pathological function. This shift from physiological to aberrant loads on the complex is hypothesized to be a significant deviation from optimum function. This is because increased joint mobility due to PDL degeneration and coronal alveolar bone support can cause increased tooth movement with the alveolar socket. Such argument can be further reinforced by the fact that the ligature had to be reintroduced every two to three days in the rats. Based on our previous work, regions that are predicted to experience increased strain during the initial disease stages include coronal attachment sites, apical compression-dominant regions, and predominantly the interradicular regions [70] (the PDL-space at the interradicular region is narrower compared to PDL-space around the tooth (Figure 5(a))). As such, it is conceivable that, within ligated groups, healthy PDL in interradicular regions, and apical to diseased regions could experience amplified strains during mastication compared to their control counterparts. Additionally, strains could be altered at the PDL-bone and PDL-cementum interfaces [71] in ligated groups. These altered three-dimensional strain profiles are of special interest because deviations from normal physiological strain can lead to pathological function (aberrant loads/function), and compromised mechanotransduction over time [5, 72, 73] including the soft-hard tissue interfaces where multiple cell types reside.

Ligated groups exhibited different patterns of morphological adaptation over time compared to control groups as indicated by changes in PDL-space. Complementary increase in RANKL expression and osteoclast activity, an increase in interradicular PDL-space (15 days, Figure 5(a)(ii)) could indicate excessive strains in the interradicular complex at a later time, resulting in resorption and shifting the adaptive effects of our model to those observed in a hyperocclusion model [74, 75]. As such, it is possible that in our study the interradicular bone commonly known as the fulcrum for tooth rotation [70] shifted, thus altering the strain field within the entire bone-PDL-cementum complex. 

The shift toward aberrant function can promote a net change in the localization and intensity of RANKL. RANKL is a mechanosensitive molecule that decreases in expression with tension in osteoblasts [76]. When comparing alveolar crests within ligated complexes, RANKL expression was seen to increase between 8 and 15 days on the distal side but decreased over time on the mesial side (Figures 2(d) and 2(e)). Although RANKL expression increases at each time point between ligated and controls both mesially and distally due to a host inflammatory response, the suggested age dominated mesial-tension due to growth may have counteracted this increase during prolonged mechanical loading. As such, while a RANKL expression increase was seen in 4-day ligated complexes compared to controls, a similar number of RANKL(+) cells were seen between ligated and control complexes at 15 days. In contrast, the progressive increase of RANKL(+) cell count from 8 days to 15 days on the distal side was most likely caused by the compounded effect of compression-induced expression due to distal drift and host inflammatory response [77, 78]. Although we correlate RANKL expression increase to osteoclast activation, it is important to note that a RANKL/osteoprotegerin (OPG) ratio should be used as the standard index for formative and resorptive bone [16]. Together, this data suggests that an early host inflammatory response drives increased biochemical expressions coronally. However, later changes in expressions of respective biomolecules due to opposing mechanobiological effects on mesial and distal sides are induced by aberrant function of the ligated joint. Aberrant function-related effects were seen in secondary cementum. In this study, the complementary data of collagen birefringence and FN expression seen at the distal secondary cementum is proposed as a compensatory adaptation due to a net increase in distal bone resorption and maintenance of age-related normal physiological activity at the mesial complex (Figure 5(c)). However, birefringence indicated by PSR is only a complementary marker to the more confirmatory FN expression. This is because collagen birefringence identified through PSR staining is also a function of section thickness and is highly dependent on fiber orientation relative to the polarizers (movies were included to highlight the specificity of our results—Figure S1 and supplemental movies) and level of tissue demineralization and collagen integrity. 

Secondary cementum is hypothesized to adapt to occlusion during the posteruption phase of tooth development and has been shown to respond to load [35, 79]. As such, it is conceivable that secondary cementum adaptation would follow coronal bone resorption as a mechanism to compensate for disruption due to inflammation. In this study, we used two identifiers to detail the adaptation of secondary cementum. These included collagen birefringence and FN expression [24, 80, 81]. The triggering of the local biochemical effects caused an increase in apical collagenous matrix organization with time (Figure 5(c)). As a result, an altered birefringence was observed at the mesial, but higher at the distal PDL-secondary cementum interface in ligated groups (Figure S1 supplemental movies). This implies that inflammation-induced mechanobiology remotely stimulated organic matrix within the apical cementum (Figures 5(c), 6, S1, and S2). Although an increased level of birefringence equivalent to that of controls was observed at 4 days, with increased time, both the width and intensity of birefringence at the distal secondary cementum surface increased. It is plausible that the complex has adapted to changes resulting from ligature-induced coronal inflammation in the tension-dominant mesial region and the compression-dominant distal region. However, adaptation manifested itself in apical regions as new bone formation, which was predominant on the mesial side, and new cementum formation on the distal side (Figures 5(c) and 6) within respective groups.

The aforementioned secondary cementum adaptation detailed through collagen birefringence can be further strengthened by FN expressions. Nonphysiological tensions at the mesial complex of ligated rats due to coronal degeneration of transseptal and periodontal fibers could have altered the FN mRNA expression and protein content apically (Figure 6) [82, 83]. The localization pattern of a wide FN expression band apically could be from an increased organization around periphery of osteo- or cementoblast cells and increased density as the collagenous matrix is generated [84]. 

FN is an important chemotactic protein for the storage of growth factors, along with its prolific interactions with cell surface molecules to facilitate cellular adhesion, migration and regulate cellular differentiation and proliferation [24]. Although FN is not a direct marker for hard-tissue formation using immunohistochemistry, it provides evidence that this multipurpose protein can amplify the response of osteoblast progenitors to alter the regenerative or reparative potential of organic matrices during mechanobiological adaptation [83]. The same may hold true for cementoblast progenitors on the tooth side, resulting in mesial deposition of secondary cementum. While compressive loads have been shown to cause a decrease in FN expression within PDL cells [82, 83], when compared to controls, the distal regions in ligated molars interestingly illustrated increased FN expression at PDL-cementum attachment sites and through the distal precementum layers of the complex (Figure 6). 

We summarize the results by presenting a biomechanical model representative of the measured downstream localization and expression level changes of mechanosensitive proteins and net spatiotemporal changes in the PDL-space (Figure S3). On the distal coronal side, temporal trends of PDL-space were directly correlated with those of osteoclastic activity. As such, the net increase in PDL-space on the distal coronal side in ligated groups could be explained by an increased osteoclastic activity due to increased compressive strains within the PDL. Increased compression could arise from increased whole body distal rotation of the tooth (Figure S3A.3) coupled with coronal inflammation. In both control and ligated groups, osteoclastic activity increased significantly at 8 days and remained significantly higher at 15 days when compared to 4-day controls. While there was greater osteoclastic activity in ligated groups at 8 days compared to the controls, the lack of significant difference between control and ligated groups at both time points could be explained by (1) development-related changes at early-time points, which may have masked the effect of inflammation and mechanobiological response, and/or (2) a need for a larger sample size. However, it is interesting to note that there was a decreased but sustained osteoclastic activity at 15 days, which could have affected the observed PDL-space. 

In the apical segment of the distal complex, the trend observed in PDL-space did not correspond to the osteoclastic activity change. Specifically, when correlating trends of osteoclastic activity and PDL-space over time, the increased osteoclastic activity did not match the decreased PDL-space in 8-day ligated joints. Furthermore, the measured increase in width of FN expression adjacent to the distal secondary cementum, which indicates new cementum apposition, calls for speculation that the PDL-space in this region experienced increased tensile strains during increased distal rotation and vertical displacement (Figure S3A.3). Therefore, the PDL-space is expected to become narrower than that of control groups over time (Figure S3A.4). However, the opposite morphological change was observed. The increase in PDL-space of ligated joints at 15 days when compared to controls and 8-day ligated joints suggests that there was a delay in observable morphological effects (resorption of bone) after the increase of osteoclast activity [69]. Namely, it can be speculated that 8 days of ligation was closer to the beginning of an increase in osteoclastic activity that carried into 15 days of ligation, resulting in an increased PDL-space. As such, the new cementum apposition labeled by FN could have been missed in micro-XCT measurements, since the FN-labeled band indicates a predominance of organic matrix. Therefore, PDL-space could be narrowed with prolonged observation as the cementum layer is given time to mineralize. Further studies identifying cementum growth using fluorochrome technique or cementum-specific markers are necessary and could be used to verify this phenomenon. Similarly, the trends in osteoclastic activity on the mesial side reflected opposite morphological changes in PDL-space at corresponding time points. 

From a morphological standpoint, the significantly reduced PDL-space in more apical, unaffected regions of ligated fibrous joints can be attributed to increased tensile strains in this area due to coronal degradation [70]. Although it is well established that experimentally induced periodontitis stimulates inflammation-induced osteoclastic activity, such activity is limited to the coronal regions [85]. Therefore, the osteoclastic activity observed in more apical regions of ligated complexes could be the manifestation of the resorption/formation coupling of physiological bone turnover [86]. However, the amplified strains could have accelerated adaptation of the complex with an increased rate of bone turnover. From a strain localization perspective, these normally compression-dominant regions are predicted to be shifted to tension-dominant regions. This is due to an apically migrating fulcrum of tooth rotation that stems from the increased mobility of the tooth as a result of coronal attachment loss and resorption of interradicular bone (Figure S3A.3). Our model is also supported by the increased band width of FN expression observed at the PDL bone attachment site at the apical region on the mesial side (Figures 6(a3), 6(b3), 6(c3), and S3A.4).

From these results, we speculate that disease-related changes promote adaptation of bone and apical cementum, which was predominantly observed on the mesial-coronal and distal-apical sides of the tooth. It is important to note that the mechanical strains within the complex of a single root are assumed to be similar to strains in the entire complex of a tooth despite the ligature. Hence, the mesiodistal effects discussed in the bone-PDL-tooth complex should be similar to those observed in the corresponding complexes of the mesial and distal roots of the same tooth.

## 5. Conclusions 

In this present study, we clarified the alteration due to disease by distinguishing disease-induced effects from physiologically determined phenomena with unpaired control groups. In conclusion, our results showed that coronal induction of an inflammatory perturbation affected the overall distribution of biochemical molecules in the entire complex. Furthermore, concomitant function imposed on the compromised complex could have accelerated tissue adaptation to meet functional demands (Figures S2 and S3). To be specific, a net decrease in functional space was identified in the mesial complex, whereas a net increase in functional space was identified in the distal complex. In addition, an adaptive response in secondary cementum, that is, predominant cementum formation, was more apparent distally. The altered expression at later time points suggests that prolonged function can manifest into excessive loads on the diseased fibrous joint, shifting physiological function into an impaired function when compared to controls. It is imperative that future studies should measure the biomechanical response of joints that have undergone short- and long-term changes to inflamed and degraded fibrous joints. When measured, the biomechanical results will validate the proposed model that early morphological changes due to disease can impair function, eventually causing the observed secondary adaptations of the fibrous joint.

## Supplementary Material

Supplementary materials include a set of supplemental movies and 3 supplemental figures. Supplemental movies include specimens rotated relative to the crossed polarizer and analyzer to illustrate varying birefringence in collagen of the PDL from controls and ligated groups. Supplemental Figure 1 includes a single micrograph illustrating birefringence of PDL taken from control and ligated specimens across time points, respectively. Supplemental Figure 2 illustrates a merged fluorescence image of various immunolabeled biomolecules investigated in this study. Supplemental Figure 3 summarizes the observed changes in biomolecular expressions as a function of anatomical location within the complex. Supplemental Figure 4 presents a hypothesis illustrating the mechaniobioliogical-induced bone and cementum adaptation and, as a result, an overall change in organ-level biomechanics. Details pertaining to movies and figures can be found within respective captions.
Supplemental Movies. Movies showing rotation of PSR stained control and ligated complexes relative to the crossed polarizer and analyzer. 360 degrees rotation of each distal and mesial complex is shown in five degree increments. (A) 4 day control distal complex, (B) 4 day control mesial complex, (C) 4 day ligated distal complex, (D) 4 day ligated mesial complex, (E) 8 day control distal complex, (F) 8 day control mesial complex, (G) 8 day ligated complex, (H) 15 day control distal complex, (I) 15 day control mesial complex, (J) 15 day ligated distal complex, (K) 15 day ligated mesial complex.
Supplemental Figure 1. Micrographs illustrating collagen birefringence differences between control and ligated bone-PDL-cementum complexes. Distal (red) and mesial (green) complexes are delineated for each control and ligated sections stained with PSR at 4, 8, and 15 days. These images at 4x magnification act as anatomical references for the supplementary movies. *Alveolar bone (AB), dentin (Den), periodontal ligament (PDL), secondary cementum (SC), transseptal fibers (TF).*
Supplemental Figure 2. Merged image of RANKL, FN, and DAPI immunofluorescence signals. An overlay of panels representing immunofluorescence for RANKL (red), FBN (green), and DAPI (blue). (A1) 4 day control mesial complex, (A2) 4 day control distal complex, (B1) 8 day ligated mesial complex, (B2) 8 day ligated distal complex, (C1) 15 day ligated mesial complex, (C2) 15 day ligated distal complex. White arrows indicate osteoclast-like cells in resorption pits at the PDL-bone interface and the alveolar bone crest. *Alveolar bone (AB), dentin (D), secondary cementum (SC), new cementum (N), and vasculature (V).*
Supplemental Figure 3. Models annotating (3A) fundamental changes in organ level biomechanics, and (3B) correlates between observed morphological changes to spatial biochemical expressions emphasizing morphological adaptations. (3A) TOP PANEL: (1) The healthy fibrous joint exhibits alveolar bone crest (ABC) height and PDL structure for normal attachment support. As such, it undergoes limited, but controlled vertical displacement, torsion, and distal tooth rotation, during occlusal loading. This causes coronal compression at the distal complex of the fibrous joint. (2) Compression-induced resorption of mineralized tissues in coronal regions of the joint is shown with orange arrows. Within physiological limits, the functional PDL-space is maintained through load-induced strains and subsequent mechanobiological activity at soft-hard tissue interfaces and bulk PDL. This results in no net change to the functional PDL-space. BOTTOM PANEL: (3) Loss of coronal attachment through inflammation-induced resorption of the ABC and PDL degradation is highlighted. Loss of attachment support causes increased vertical tooth displacement and whole body rotation. Resorption of interdental and interradicular AB lowers the fulcrum, altering the whole body rotation of the tooth. Organ-level biomechanics prompts increased compressive loads at the distal coronal complex, along with increased tensile loads at the mesial coronal and mesial apical PDL-spaces and distal PDL-secondary cementum interface. (4) Perpetuating function (mastication) causes a shift in strains at soft-hard tissue interfaces, promoting net mineralized tissue formation (blue arrows, green line) in areas experiencing tensile strains and net mineralized tissue resorption (red arrows) in areas experiencing compressive strains. The net mineral-forming and -resorbing events cause large scale morphological changes in the bone-PDL-tooth complex that ultimately potentiates the joint towards disease progression and subsequent failure. (3B) Schematic drawing shows changes in the expression pattern of target biomolecules, including RANKL (red), TNF-*β* (brown), FN (green), TRAP (red), PSR (orange), and PDL-space (gray), of ligated groups compared to control groups.
