# Supplementary material for: The Adaptive Nature of the Bone-Periodontal Ligament-Cementum Complex in a Ligature-Induced Periodontitis Rat Model
Source: Biomed Res Int. 2013 Jul 2;2013:876316. doi: 10.1155/2013/876316 (PMC3713652; doi:10.1155/2013/876316)
Supplement: Supplementary file 9 [file 876316.f9.pdf]

Supplemental Figure 1

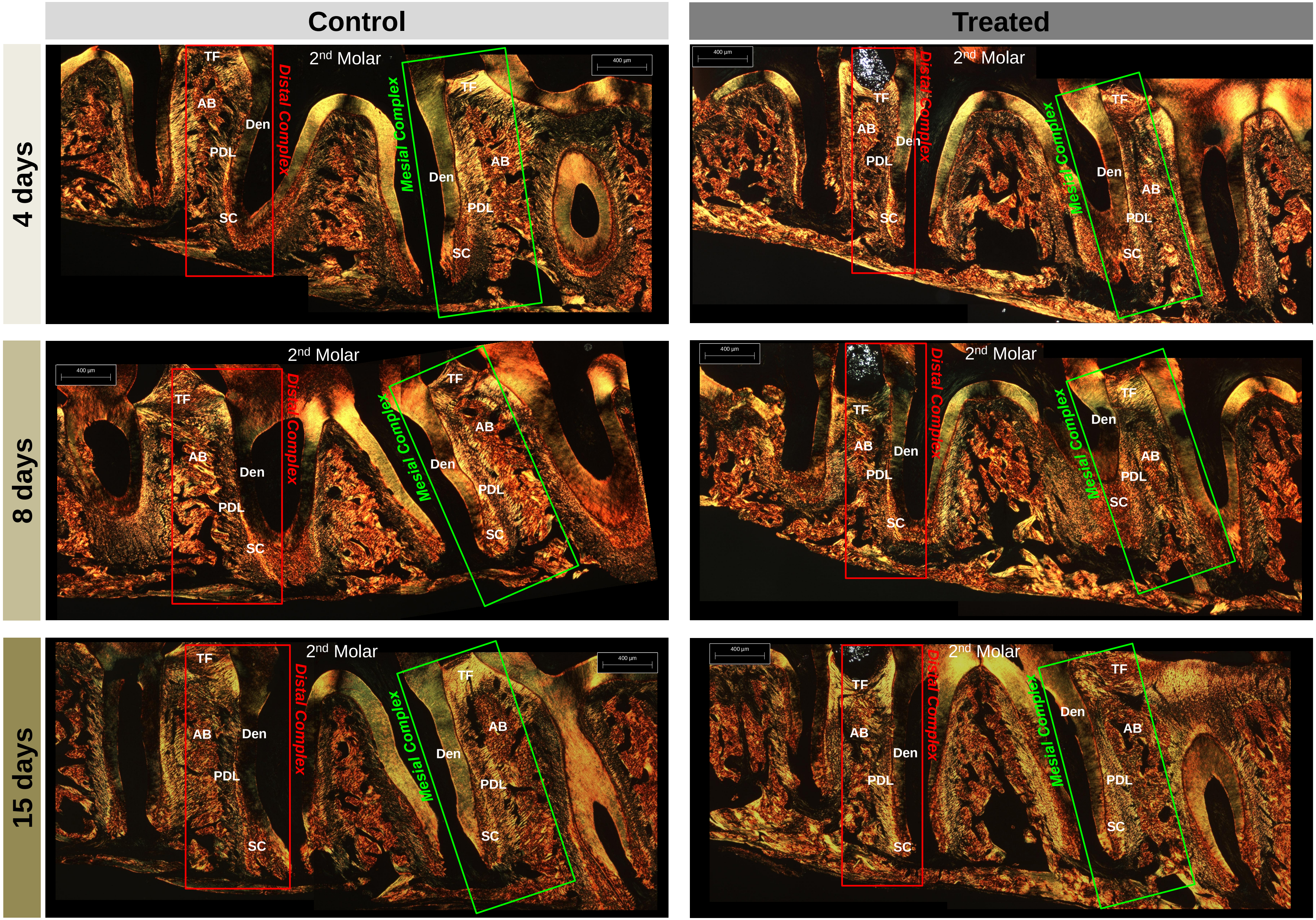

Supplemental Figure 2

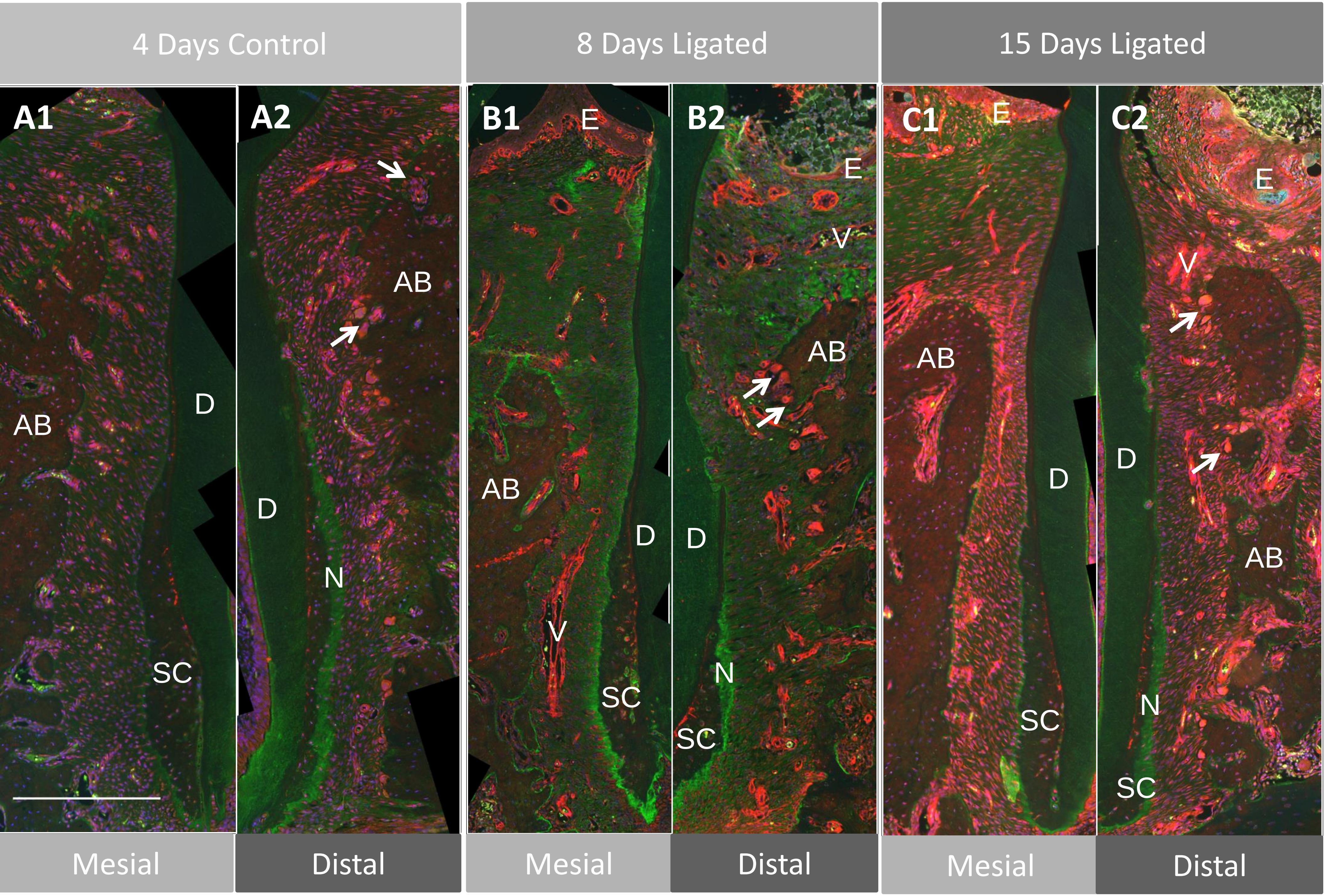

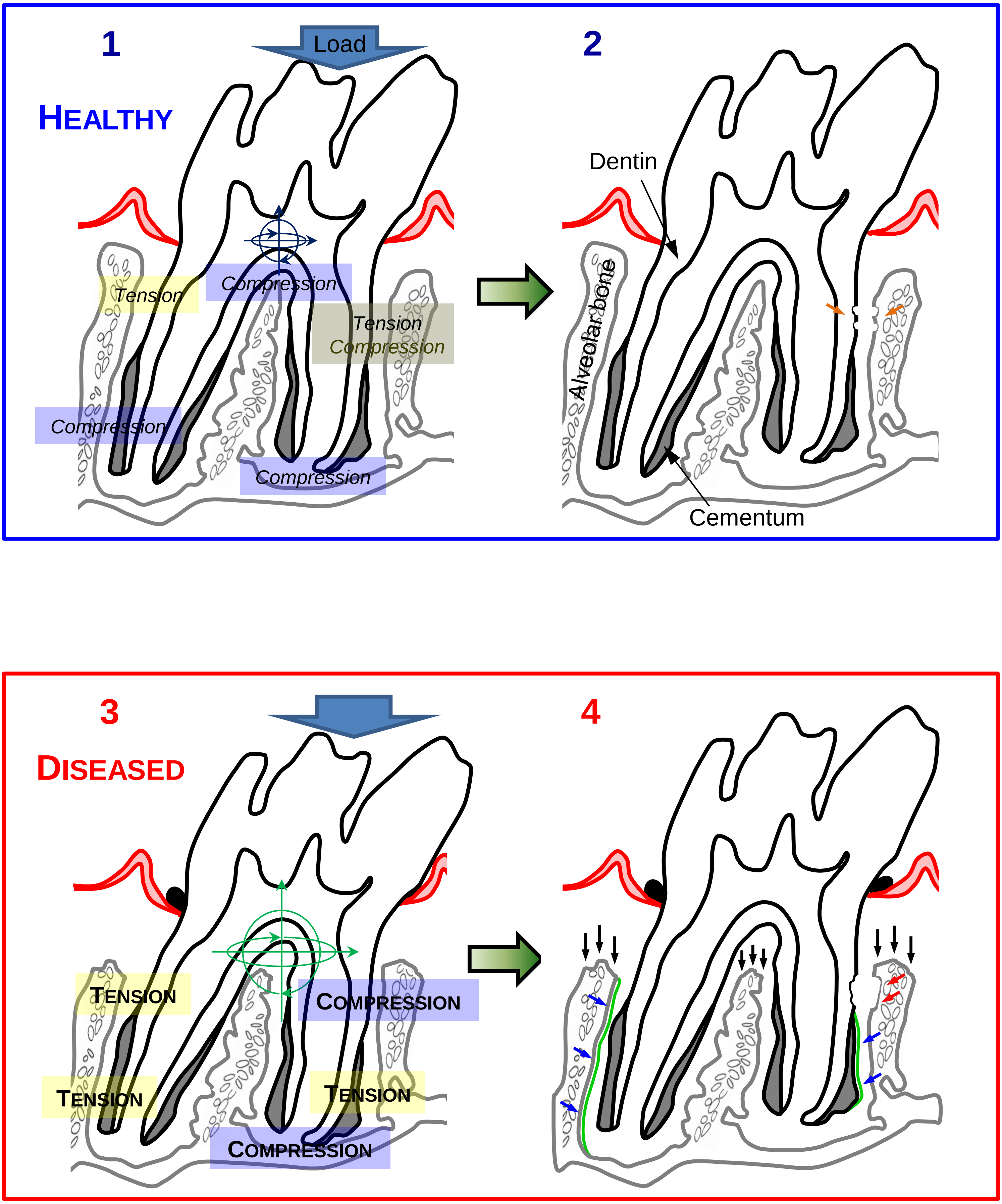

Supplemental Figure 3A

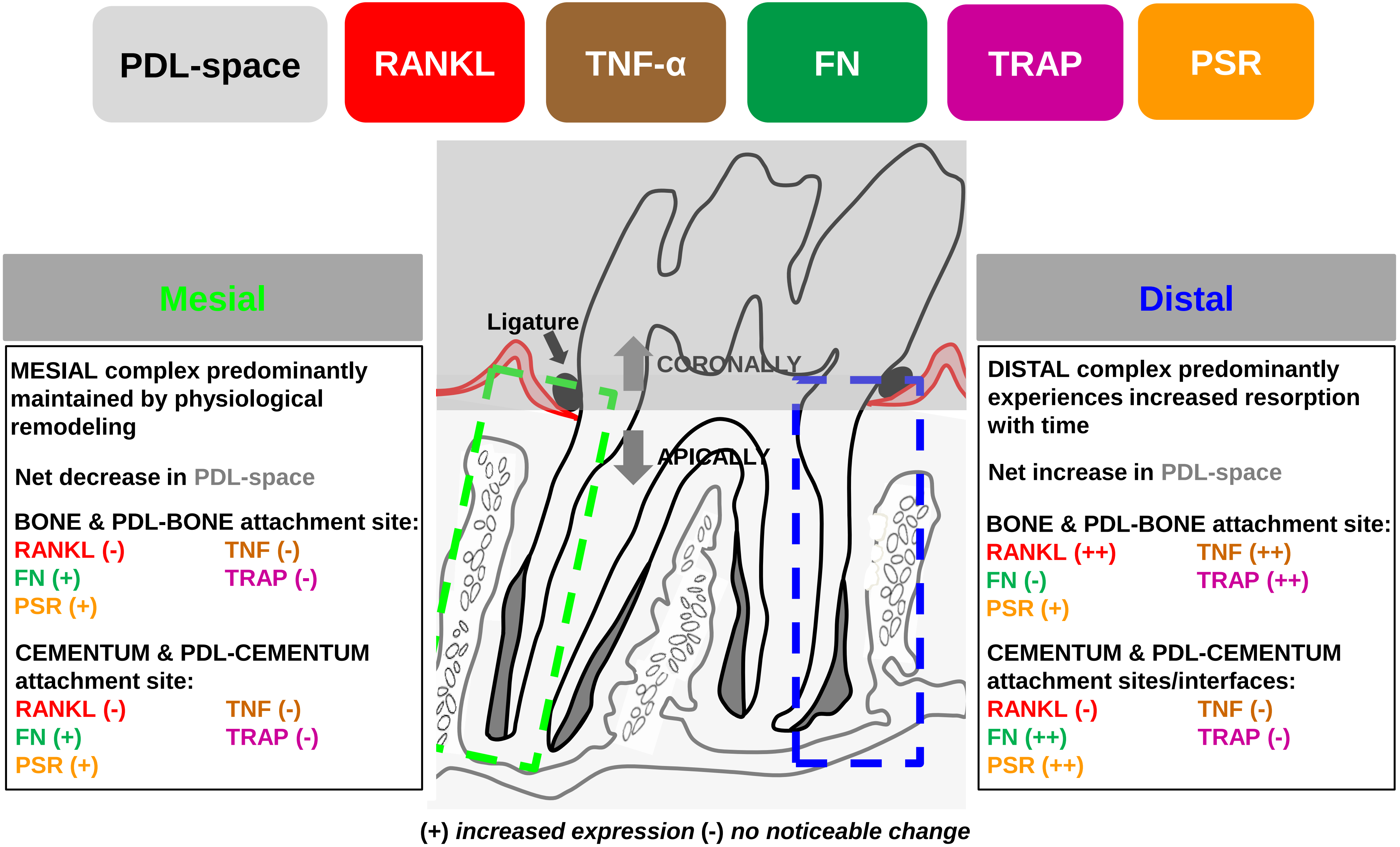

Supplemental Figure 3B
